# Association of *CD58* polymorphism and multiple sclerosis in Malaysia: a pilot study

**DOI:** 10.1186/s13317-019-0123-7

**Published:** 2019-12-17

**Authors:** Yee Ming Ching, Shanthi Viswanathan, Nurhanani Mohamed Nor, Shuwahida Shuib, Balqis Kamarudin, Salawati Mansor, Ainur Yusniza Yusof, Masita Arip

**Affiliations:** 1 Autoimmune Unit, Allergy and Immunology Research Centre, Institute for Medical Research, National Institute of Health, Selangor, Malaysia; 2 Neurology Department, Hospital Kuala Lumpur, Kuala Lumpur, Malaysia; 3 Allergy and Immunology Research Centre, Institute for Medical Research, Block C6, National Institute of Health Complex, No. 1, Jalan Setia Murni U13/52, Seksyen U13, Bandar Setia Alam, 40170 Shah Alam, Selangor Darul Ehsan Malaysia

**Keywords:** Multiple sclerosis, *CD58* polymorphism, Single nucleotide polymorphism

## Abstract

**Background:**

Multiple sclerosis is an immune mediated disease targeting the central nervous system. Association of non-human leukocyte antigen gene, *CD58*, with multiple sclerosis has been reported in several populations but is unclear among Southeast Asians. This pilot study was conducted to explore the association between *CD58* polymorphism and multiple sclerosis among the Malay population in Malaysia.

**Methods:**

Blood samples were collected from 27 multiple sclerosis patients, and compared with 58 age- and gender matched healthy individuals. All patients were tested negative for anti-aquaporin 4. DNA was extracted from the blood and genotyped for 3 single nucleotide polymorphisms rs12044852, rs2300747 and rs1335532 of gene *CD58* by real-time PCR.

**Results:**

The majority of multiple sclerosis patients were female (85.2%). The general mean age of onset was 30.5 years. Genotyping results showed that frequencies of the alleles were between 40 and 50% for MS patients and healthy individuals. Association (allelic model) between multiple sclerosis and *CD58* gene polymorphism alleles rs12044852 (p = 0.410), rs2300747 (p = 0.881) and rs1335532 (p = 0.407) were indistinct.

**Conclusions:**

The impact of the *CD58* gene polymorphism was not prominent in this pilot study, implying that genetic composition contributing to multiple sclerosis may be different between different populations, thus results in a heterogeneity of disease manifestation and distribution.

## Background

Multiple Sclerosis (MS) is an immune-mediated disease characterized by inflammation and demyelination of the central nervous system (CNS). MS patients can present with a broad spectrum of neurological symptoms such as visual loss, muscle weakness, sensory loss, incoordination, cognitive dysfunction and bladder problems [1]. A systemic analysis of MS has reported that age-standardized prevalence was greater than 120 cases per 100,000 population in North America and some northern European countries, moderate (60–120 per 100,000) in some countries in Europe and Australasia, and lower in Africa, Asia and northern South America region (5 per 100 000) [2, 3]. In Malaysia, the prevalence of MS was estimated to range from 2 to 3 per 100,000 [4, 5].

The development of MS is commonly associated with the interaction between genetic susceptibility and environmental factors. Genetic association of MS, especially the variation in human leukocyte antigen (HLA) location on chromosome 6, has been generally considered as the highest risk for the disease development [6]. However, the influence of the *HLA* gene alone is insufficient to fully explain the role of genetic in the pathogenesis of the disease. A genome wide association study of MS patients among the United States (US) and United Kingdom (UK) was performed by the International Multiple Sclerosis Genetics Consortium (IMSGC) in 2007. They found that several single nucleotide polymorphisms (SNPs) from the non-HLA region were highly associated with MS [7]. One of the MS related non-HLA genes is *CD58*.

The *CD58* gene is located on chromosome one and it encodes a member of the T lymphocytes CD2 protein ligand, which plays an important role in signal transduction in T cell activation [8, 9]. Regulation of T cells is crucial in maintaining the body’s immune response and tolerance towards self and foreign antigens. Failure of immune tolerance towards self-antigens results in autoimmunity. The *CD58* SNPs have been studied in European ancestry [10, 11] but little is known about their association with MS in Asian, especially in Southeast Asian. Therefore, in this study, we aimed to explore and investigate the association of several *CD58* SNPs and MS in the Malay population in Malaysia.

## Methods

### Subjects of study

Samples for this study consisted of 27 MS patients, who were recruited from the Neurology Clinic, of Hospital Kuala Lumpur. This study enrolled patients of Malay ancestry and were diagnosed with Multiple Sclerosis (MS) by a neurologist based on the revised McDonald criteria of 2017 [12]. Clinical subtypes of the disease included relapsing–remitting MS (RRMS) and secondary progressive MS (SPMS). Demographic data and characteristic of patients such as duration of disease, age onset, MRI results (infratentorial lesion and juxtacortical lesion) and Expanded Disability Status Scale (EDSS) scores were collected. All samples were tested for anti-aquaporin 4 antibodies using commercially available kit (Euroimmun, Lubeck, Germany). Patients with positive anti-aquaporin 4 antibodies were excluded from the study. The control group comprised 58 biological unrelated individuals of the same ethnic background and similar age. Informed consent was obtained from all patients and control individuals participating in this study and their anonymity was preserved. This study was approved by the Medical Research and Ethics Committee of Malaysia Ministry of Health (NMRR-13-1029-18067).

### Sample preparation and genotyping

DNA was extracted from blood samples according to the standard method using the commercial DNA extraction kit (Qiagen, Germany). Three SNPs (rs12044852, rs1335532 and rs2300747) in *CD58* gene were selected based on findings of genome wide association studies (GWAS) and were reported to be highly associated with MS [7, 11, 13]. The 3 SNPs were genotyped for all study subjects and control using Taqman assay (Applied Biosystems, USA): Taqman SNP Genotyping assay C_31433800_10 (rs12044852), C_15755405_10 (rs2300747) and C_8700717_10 (rs1335532), on the ABI 7500 Fast Real-time PCR system (Thermo Fisher Scientific, USA).

### Statistical analysis

Statistical analysis was performed using IBM SPSS Statistics version 22 for Windows (IBM Corp, USA). Allele frequencies for MS patients and healthy controls were calculated and compared to healthy controls. The association between *CD58* polymorphism and MS patients’ characteristics was analysed using Chi square or two-tailed Fisher’s exact test, odd ratios (OR) and 95% confidence interval (95% CI) were calculated. Value of p ≤ 0.05 was considered statistically significant.

## Results

Our study population consisted of 27 Malay MS patients, predominantly female, with female to male ratio of 5.8:1. Control groups comprised 52 females and 6 males of healthy individuals. The demographic and clinical features of the study population are shown in Table 1. Majority of MS patients in this study population were diagnosed as RRMS (81.5%).

**Table 1:** Demographic and clinical features of study subjects

Variables	Controls, N (%)	MS patients, N (%)
Female	52 (89.7)	23 (85.2)
Male	6 (10.3)	4 (14.8)
Age of onset, mean ± SD (range)		30.5 ± 10.3 (16–56)
Age of time of analysis, mean ± SD (range)	42.3 (26–65)	36.5 ± 11.7 (20–62)
Disease type		
Relapsing remitting MS (RRMS)		22 (81.5)
Secondary progressive MS (SPMS)		5 (18.5)

Allelic prevalence for each of the *CD58* SNPs studied shows a range of 40 to 50% for each allele in both MS patients and controls (Table 2). Frequencies of *CD58* haplotypes for this study population are shown in Table 3. Table 4 shows the association analysis of the *CD58* SNP alleles rs12044852 (p = 0.410), rs1335532 (p = 0.881) and rs2300747 (p = 0.407) between MS patients and controls. Analysis of the results revealed no significant association between *CD58* SNPs and age of onset, MRI parameters and disease activity. *CD58* SNPs were also not significantly different between types of MS.

**Table 2:** Genotype distribution and allele frequency *CD58* SNPs in MS patients and controls

SNPs	Genotypes/Alleles	MS (%)	Controls (%)
Rs12044852	AA	18.5	31.0
	AC	55.6	46.6
	CC	25.9	22.4
	A	46.3	54.3
	C	53.7	45.7
Rs1335532	GG	25.9	39.7
	GA	63.0	37.9
	AA	11.1	22.4
	G	57.4	58.6
	A	42.6	41.4
Rs2300747	AA	18.5	20.7
	AG	59.3	41.4
	GG	22.2	37.9
	A	48.1	41.4
	G	51.9	58.6

**Table 3:** Frequency of *CD58* haplotypes in this study

Haplotype (Ht)	Rs1335532	Rs2300747	Rs12044852	Frequency
Ht1	G	G	A	0.511
Ht2	A	A	C	0.405
Ht3	G	G	C	0.047
Ht4	G	A	C	0.024
Others				0.013

**Table 4:** Allelic analysis of *CD58* SNPs in MS patients and control

SNPs	Alleles	p value	OR	95% CI
RS12044852	A,C	0.410	0.7	0.4–1.4
RS1335532	G,A	0.881	1.1	0.5–2.0
RS2300747	A,G	0.407	1.3	0.7–2.5

## Discussion

Single nucleotide polymorphisms (SNPs) are the most common type of DNA polymorphisms in human and their relations with autoimmune diseases such as MS are noteworthy. Association of SNPs in non-HLA genes and autoimmune diseases has been reported in various studies involving the US and European population in the last decades [14, 15].

For MS, one of the candidate non-HLA genes reported to be associated with the disease is *CD58* [16]. It is suggested that alteration of *CD58* expression affects the regulation of FoxP3, a protein highly expressed in regulatory T cells (Treg), leading to functional changes in Treg cells and influence the body’s immune response against its own cells. Dysfunction or reduction of Treg has been reported in various studies of autoimmune diseases including MS [17]. The expression of CD58 mRNA has been shown to be increased significantly in samples collected from MS patients during remission than those sampled during relapse [13].

The demographic characteristic of this study is in agreement with findings of other studies. Age of onset in this study population ranged from 16 to 56 years old, with mean of 30.5 ± 10.3 years which falls within the range of findings in other studies around the region: 28.6 ± 9.9 years in another study in Malaysia [18] and 32.7 ± 11.5 years in Thailand [19]. In South India, the age of onset was noted at 38.3 ± 12.8 years [20]. Out of 27 MS patients in this study, about 85.2% were females and 14.8% were males, with female to male ratio of 5.8:1. The ratio is consistent with an analysis of data from the Danish Multiple Sclerosis Registry that showed the incidence in women doubled between 1950 and 2009, while the increases among men were relatively moderate [21]. The rapid change in incidence among women might be due to environmental changes such as the rise in obesity, increased cigarette smoking, and changes in the frequency of breastfeeding infants [21, 22].

In this pilot study, we investigated the possible association of *CD58* SNPs, namely rs2300747, rs12044852 and rs1335532, with MS amongst Malay population in Malaysia. Our results showed that the frequency of rs2300747G was higher in the control group compared to the MS patients, suggesting a potential of a protective value in rs2300747G, although the difference was statistically insignificant. A study done by De Jager et al. indicated that rs2300747G was a protective allele associated with MS and that East Asian cell lines expressed higher frequencies of rs2300747G compared to the cell lines of Europe ancestry, which might explain a lower frequency of MS amongst Asians [11]. This is also supported by a meta-analysis done by Liu et al. [23] which reported polymorphism of rs2300747G as a protective allele against MS. However, findings of SNP rs2300747 and MS were inconsistent in different populations. Like our results, no evidence of protective allele in SNP rs2300747 was found in a study on the association of non-MHC region and MS among Indian population in India [24].

In a replication GWAS study conducted by The Australia and New Zealand Multiple Sclerosis Genetics Consortium (ANZgene) which analysed more than 1500 MS cases, it was reported that SNP rs1335532 of *CD58* is one of the non-HLA SNPs that was highly associated with MS [13]. However, the same SNP has been reported to be significantly associated with neuromyelitis optica spectrum disorders (NMOSD), another spectrum of inflammatory demyelinating disease, in a case–control study of Koreans [25, 26]. On the other hand, the other *CD58* SNPs, rs12044852, has been repeatedly reported to be associated with MS [27, 28]. A case–control study involving 200 MS patients and 200 healthy controls in a subset of Iranian population reported that the rs12044852 polymorphism of *CD58* has a profound effect on the onset of MS but not significantly associated with patients’ gender, disease severity and type of MS [29]. Our results showed that the rs12044852 polymorphism was not significantly associated with MS. This is also similar to a study focused on genetic susceptibility to MS in African Americans, that showed rs12044852 and rs2300747 were not significantly associated with MS [30].

There were a number of limitations in our study which possibly restricted the analysis of the results and affected the outcome of the study. Compared with other studies on SNPs [13, 31], the number of patients and controls recruited in this study was relatively small for genetic association study and may not represent the true population. Small sample size also has less statistical power and could introduce bias to this study, leading to insignificant results. Although a larger sample size could increase statistical power, it could also be costly and takes longer time frame to complete the sample collection due to the relatively low frequency of autoimmune diseases around the region. Another option that can be considered to improve this study is by increasing the sample size of the control to 1:4 case–control ratio if the assumptions of model can be fulfilled [32]. Another limitation in this study that can be improved is the location of sample collection. Due to location of the hospital, patients recruited in this study were mainly from the central urban region of Peninsular Malaysia. This could introduce bias as the Malay population in Malaysia can be clustered into a few sub-ethnic groups that could be genetically different [33]. In addition to that, like other autoimmune diseases, the development of MS involves a complex interaction between genetic susceptibility and environmental factors. *CD58* gene may play an indirect role in disease development and the differences in other genetic components of our study population may have contributed to the outcome.

In conclusion, our exploratory study did not show significant association between *CD58* SNPs (rs12044852, rs1335532 and rs2300747) and MS in our study population. However, further investigations with optimal sample size, more genetic markers and correlation with environmental factors are necessary to help shed some light on this disease in our country.

## Data Availability

The datasets used and/or analysed during the current study are available from the corresponding author on reasonable request.

## Abbreviations

MS: Multiple sclerosis
HLA: human leukocyte antigen
SNPs: single nucleotide polymorphisms
RRMS: relapsing–remitting MS
SPMS: secondary progressive MS
EDSS: Expanded Disability Status Scale
